# Exploring the Antibiofilm Effect of Sertraline in Synergy with *Cinnamomum verum* Essential Oil to Counteract *Candida* Species

**DOI:** 10.3390/ph17091109

**Published:** 2024-08-23

**Authors:** Alexia Barbarossa, Antonio Rosato, Antonio Carrieri, Luciana Fumarola, Roberta Tardugno, Filomena Corbo, Giuseppe Fracchiolla, Alessia Carocci

**Affiliations:** 1 Department of Pharmacy—Pharmaceutical Sciences, University of Bari “Aldo Moro”, 70125 Bari, Italy; antonio.rosato@uniba.it (A.R.); antonio.carrieri@uniba.it (A.C.); roberta.tardugno@uniba.it (R.T.); filomena.corbo@uniba.it (F.C.); giuseppe.fracchiolla@uniba.it (G.F.); 2 Interdisciplinary Department of Medicine, School of Medicine, University of Bari “Aldo Moro”, 70124 Bari, Italy; luciana.fumarola@uniba.it

**Keywords:** drug repositioning, antimicrobial resistance, synergism, checkerboard microdilution method, antifungal effect

## Abstract

The emergence and spread of drug-resistant pathogens, resulting in antimicrobial resistance, continue to compromise our capability to handle commonly occurring infectious diseases. The rapid global spread of multi-drug-resistant pathogens, particularly systemic fungal infections, presents a significant concern, as existing antimicrobial drugs are becoming ineffective against them. In recent decades, there has been a notable increase in systemic fungal infections, primarily caused by *Candida* species, which are progressively developing resistance to azoles. Moreover, *Candida* species biofilms are among the most common in clinical settings. In particular, they adhere to biomedical devices, growing as a resilient biofilm capable of withstanding extraordinarily high antifungal concentrations. In recent years, many research programs have concentrated on the development of novel compounds with possible antimicrobial effects to address this issue, and new sources, such as plant-derived antimicrobial compounds, have been thoroughly investigated. Essential oils (EOs), among their numerous pharmacological properties, exhibit antifungal, antibacterial, and antiviral activities and have been examined at a global scale as the possible origin of novel antimicrobial compounds. A recent work carried out by our research group concerned the synergistic antibacterial activities of commercially available and chemically characterized *Cinnamomum verum* L. essential oil (*C. verum* EO) in association with sertraline, a selective serotonin reuptake inhibitor whose repositioning as a non-antibiotic drug has been explored over the years with encouraging results. The aim of this work was to explore the synergistic effects of *C. verum* EO with sertraline on both planktonic and sessile *Candida* species cells. Susceptibility testing and testing of the synergism of sertraline and *C. verum* EO against planktonic and sessile cells were performed using a broth microdilution assay and checkerboard methods. A synergistic effect was evident in both the planktonic cells and mature biofilms, with significant reductions in fungal viability. Indeed, the fractional inhibitory concentration index (FICI) was lower than 0.5 for all the associations, thus indicating significant synergism of the associations with the *Candida* strains examined. Moreover, the concentrations of sertraline able to inhibit Candida spp. strain growth and biofilm formation significantly decreased when it was used in combination with *C. verum* EO for all the strains considered, with a reduction percentage in the amount of each associated component ranging from 87.5% to 97%.

## 1. Introduction

*Candida* species are opportunistic pathogenic microorganisms that can cause systemic infections with a high mortality rate in immunocompromised and at-risk patients, thus representing a serious threat to public health [1]. Indeed, *Candida* spp. are the primary cause of 50–70% of systemic fungal infections, and candidemia is the most common hospital infection, representing approximately 15% of bloodstream infections [2]. *Candida albicans* is the most commonly isolated pathogenic species. However, other species, such as *Candida glabrata, Candida tropicalis*, *Candida parapsilosis*, *Candida krusei*, *Candida famata, Candida guilliermondii*, and *Candida lusitaniae*, have increasingly been isolated, principally in human immunodeficiency virus (HIV)-infected individuals [3,4]. According to some studies, *Candida* strains are able to adhere to artificial and mucosal surfaces to produce biofilms, incredibly complex structures [5]. Biofilm formation is a significant virulence factor that lengthens hospital stays, raises death rates, and increases the cost of antifungal therapy in patients affected by this condition [6]. In addition, the growing resistance of yeast to antifungal drugs is one of the main issues among researchers and clinicians. The biological mechanisms that decrease the sensitivity of yeast biofilms to antifungal compounds, including the most commonly used azoles, involve active drug efflux, the limited penetration of molecules through the extracellular matrix, the low metabolic activity of cells in mature biofilms, and the higher expression of various genes in biofilms than in planktonic forms [7,8]. In spite of the harmful effects fungi pose to human health, there are only a few classes of antifungal drugs currently accessible for addressing these potentially life-threatening infections; furthermore, those currently available do not completely provide safe and secure protection [9]. To overcome this issue, numerous pharmaceutical compounds from different therapeutic classes are being evaluated as antimicrobials in the drug repositioning approach to finding new, cost-effective solutions [10]. Non-antifungal medications exhibiting antifungal properties are proven to be effective in combating fungal infections. Certain antidepressant medications have demonstrated antimicrobial effects, with the most potent activity observed in the third-generation antidepressants referred to as selective serotonin reuptake inhibitors (SSRIs), such as fluoxetine, paroxetine, and sertraline [11]. Notably, sertraline, owing to its antimicrobial potential, has been shown to augment the efficacy of various antibiotics, reverse pathogen multidrug resistance, and render them susceptible to previously ineffective drugs [12,13]. Antifungal activity of sertraline has been recently demonstrated against planktonic forms and biofilms of some fungal strains [14,15]. In addition, a recent study demonstrated the synergistic activity of SSRIs, including sertraline, in combination with azoles against *Cryptococcus* spp. [16]. Another strategy for addressing therapeutic failures in the treatment of fungal infections is the use of plant-derived compounds [17,18,19]. Amongst phytochemicals, essential oils (EOs), yielded by aromatic plants as secondary metabolites, and their bioactive pure compounds have demonstrated an extensive array of noteworthy biological activities, including antibacterials and antifungals [20,21]. EOs can mitigate microbial growth and inhibit biofilm formation through distinct mechanisms associated with the breakdown of the bacterial cell wall, their influence on the breakdown of enzymes or membrane proteins, or the release of cell contents following cytoplasmic membrane failure [22,23]. The intricate composition of EOs can play a fundamental role in counteracting microbial resistance since it can be challenging for pathogens to develop resistance to several substances [24]. This suggests that EOs and antibiotics may work synergistically to combat microbial infections. This aspect matters since it may contribute to lowering the dosage and usage of antibiotics in treatments, thereby decreasing their adverse effects [25]. Among the essential oils with pronounced antimicrobial activities emerge those derived from species within the genus *Cinnamomum* (Lauraceae), including *Cinnamomum verum*, commonly referred to as Ceylon cinnamon, or the cinnamon tree [26]. Despite significant variations in the chemical composition observed across oils from different regions worldwide [27,28], *trans*-cinnamaldehyde predominates as the major constituent, constituting 47–71% of the composition, while their other constituents include eugenol and linalool. The essential oil of *C. verum* has been used for centuries in various cultural practices due to its medicinal properties. These include its use as an antiseptic, an anti-inflammatory [29], and a preservative agent [30]. Diverse studies have reported the potent antibacterial and antifungal properties of *C. verum* EO, both independently and in conjunction with antibiotics [31,32]. Indeed, a recent study reported the efficacy of low concentrations of *C. verum* EO in the inactivation of *E. faecalis* and *S. aureus* in carrot juice and white mulberry juice [33]. Furthermore, the efficacy of *C. verum* leaf essential oil has been demonstrated as a therapeutic alternative for *Candida* biofilm infections, highlighting its potential in addressing fungal resistance issues [28]. This work is part of our ongoing research program directed toward the development of new strategies to overcome antimicrobial resistance. Indeed, in recent years, we have concentrated our efforts on examining the possible synergies between EOs and antibiotics (gentamicin, oxacillin, and norfloxacin) or repurposed drugs (diclofenac, sertraline, etc.), proving the effectiveness of these combinations and raising the possibility of novel therapeutic applications [34,35,36]. In our previous work, we demonstrated the synergistic antibacterial effect on planktonic cells of sertraline in combination with *C. verum* EO [37]. Considering the compelling findings acquired and acknowledging the documented capability of sertraline to combat fungal infections and hamper biofilm growth [38,39], the goal of this work was to evaluate the synergic action of the combination of sertraline and *C. verum* EO on planktonic cells of different *Candida* strains and their ability to inhibit the growth of biofilms produced by fungal strains.

## 2. Results

### 2.1. Cinnamomum verum EO’s Chemical Composition

Gas chromatography–mass spectrometry analyses of the EO were performed following the procedure described in our previous papers [35,37]. The analysis resulted in the identification of 99% of the whole mixture. The principal constituents of *C. verum* EO are (*E*)-cinnamaldehyde (72%), linalool (6.78%), eugenol (6%), cinnamyl acetate (5%), and eucalyptol (1.47%). The other constituents are present at quantities of less than 1%, i.e., p-cymene, humulene, *a*-pinene, etc. Our results, shown in Table 1, are basically in agreement with what has been previously reported [37].

### 2.2. Antifungal Activity of Sertraline in Association with Cinnamomum verum EO against Planktonic Cells of Candida *spp.*

The synergistic antifungal effect of *C. verum* EO in combination with sertraline against planktonic cells of several strains of *Candida* spp. has been explored by means of the microdilution checkerboard technique. Table 2 displays the minimal inhibitory concentration (MIC) values for sertraline and *C. verum* EO. MICo represents the MIC value of a single component tested alone, while MICc is the MIC value of each component in the association at the most effective inhibition growth. The fractional inhibitory concentration (FIC) is calculated by the MICc/MICo ratio, and the fractional inhibitory concentration index (FICI), a parameter that expresses the synergism of the two compounds, is determined by adding the FIC of sertraline to the FIC of *C. verum* EO. In the final column of Table 1, R% describes the percentage decrease in the quantity of each combined component compared to each single component. All the FICI values were significantly lower than 0.5, in the range of 0.08–0.16 and thus denoting a marked synergism between *C. verum* EO and sertraline against all the fungal strains tested. It is worth noting that the MICc value for sertraline is markedly reduced when combined with the EO since it was found to be up to 40-fold lower than the MICo for most of the yeast species under study. Interestingly, the best results were achieved against *C. albicans* ATCC 90028 and *C. kefyr* ATCC 204093, for which the MIC values of sertraline decreased from 32.0 (MICo) to 0.80 (MICc) µg/mL and from 64.0 (MICo) to 3.20 (MICc) µg/mL, respectively, by showing a noteworthy reduction percentage of 97% in the first case and 95% in the second, as proven by the FICI values of 0.08 for both species. Concerning the American Type Culture Collection (ATCC) strains, the synergistic activity of the combination against *C. albicans* ATCC 10231 and *C. glabrata* ATCC 15126 should be noted. Indeed, a substantial reduction in the sertraline concentration is observed since it switches from 64.0 (MICo) to 3.20 (MICc) µg/mL for both strains, exhibiting a 20-fold reduction. An impressive synergistic effect was also observed on *C. albicans* A18 and *C. krusei* 31A29, both derived from clinical isolation, for which the sertraline concentrations decreased from 128.00 to 6.40 and from 64.00 to 3.20 µg/mL, respectively. Interestingly, the combination under study was also shown to be effective against *C. albicans* 10 A12 (FICI = 0.17), *C. krusei* ATCC 6258 (FICI = 0.16), and *C. tropicalis* ATCC 750 (FICI = 0.16). Overall, the clear-cut synergic activity of sertraline and *C. verum* EO determined a significant reduction percentage for the amount of each component in association with respect to the single component that ranged between 87.5% and 97%.

### 2.3. Inhibitory Effects on Candida *spp.* Biofilm Growth of Sertraline in Association with Cinnamomum verum EO

The encouraging results obtained on the planktonic cells prompted us to investigate the potential inhibitory activity of the association between sertraline and *C. verum* EO against the growth of biofilms produced by the yeast strains considered in this research. The effects of this association, obtained by means of an XTT assay, expressed as percentage values of the reduction in biofilm growth, along with the amounts of each component in association at the most effective growth inhibition, are reported in Table 3. In addition, all of the sessile minimal inhibitory concentration values (sMIC_50_) for sertraline and *C. verum* EO were reported for comparison. The combination of sertraline with *C. verum* EO was effective in hampering biofilm growth of all the *Candida* spp. tested, with the inhibition percentages ranging from 59.90 to 86.44%, thus confirming the trend of the results obtained on the planktonic cells. Indeed, the FICI values for this association were between 0.16 and 0.35, demonstrating an evident synergistic effect even on the sessile cells. Particularly interesting are the data obtained against *C. albicans* ATCC 90028 since the association of sertraline with *C. verum* EO strongly succeeded in inhibiting the biofilm growth produced by this strain, with a percentage value of 86.44% (FICI = 0.23). The combination also proved to be quite promising in preventing the growth of the biofilms formed by the strains from clinical isolation of *C. albicans* A 18 (FICI = 0.35) and *C. albicans* 10 A12 (FICI = 0.23), with percentages of 77.02 and 75.29%, respectively, and those formed by *C. albicans* ATCC 10231 (FICI = 0.16), with a percentage of 72.8%. As already observed for the planktonic cells, an interesting result was also achieved with sessile cells of *C. kefyr* ATCC 204093. Indeed, the biofilm formed by this yeast decreased with a percentage amounting to 79.80% (FICI = 0.20). The *C. glabrata* ATCC 15126, *C. krusei* ATCC 6558, *C. krusei* 31 A29, and *C. tropicalis* ATCC 750 biofilms treated with the sertraline–*C. verum* EO association also appreciably decreased, with percentages amounting to 59.90, 64.90, 69.44, and 68.25%, respectively. Another parameter that allows us to confirm the effectiveness of the association between these two substances is the significant reduction in the amounts of sertraline able to inhibit biofilm growth when combined with *C. verum* EO with respect to the amounts of sertraline tested alone. Indeed, the sMIC_50_ of sertraline ranged between 640.00 and 40.00 µg/mL, whereas the concentration of sertraline in association, for the most effective combination, ranged between 32.00 and 4.00 µg/mL, thus decreasing 10 to 20 times. This decrement was particularly pronounced for *C. glabrata* ATCC 15126 and *C. kefyr* ATCC 204093, whereby the sMIC_50_ of sertraline dropped from 320.00 to 16.00 µg/mL for both, and for *C. tropicalis* ATCC 750, for which the concentration required to hamper biofilm growth declined from 640.00 to 32.00 µg/mL.

### 2.4. Molecular Modeling Studies

**Figure 1:**
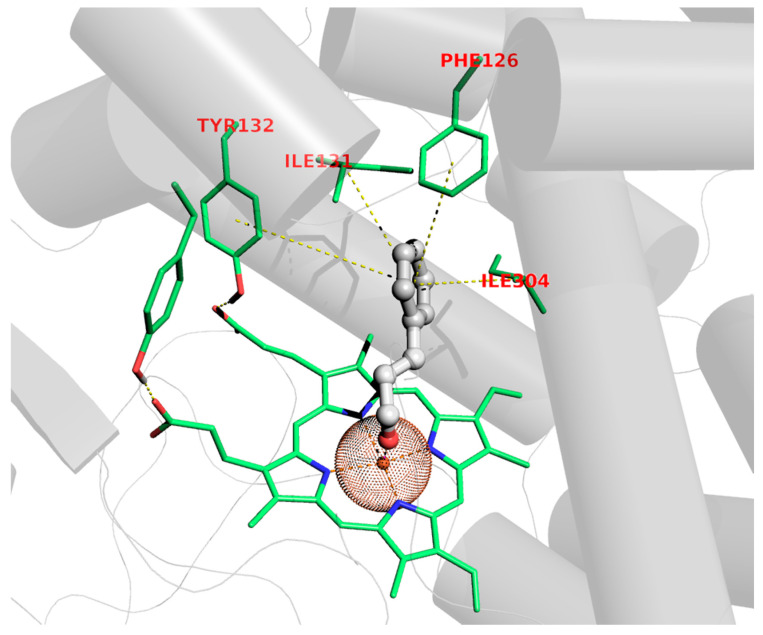
Binding pose of (*E*)-cinnamaldehyde to CYP51.

Although the antifungal properties of *C. verum* EO have not yet been related to a specific mechanism of action or related to exact targets, the *C. albicans* cytochrome P450-dependent lanosterol 14α-demethylase (CYP51) was enrolled to study the potential mechanisms of *C. verum*’s main component through molecular modeling experiments. Lanosterol 14α-demethylase (CYP51) is a member of the cytochrome P450 superfamily which catalyzes the oxidative removal of the 14α-methyl group of lanosterol to give ∆^14,15^-desaturated intermediates in ergosterol biosynthesis [44]. The selection of CYP51 as a plausible site of action is also supported by the evidence that azoles (i.e., fluconazole and miconazole) and other drugs (i.e., benzothiazines and benzoxazine) [45,46,47,48,49,50,51,52,53,54,55,56,57] inhibit cell growth upon ergosterol depletion as determined by CYP51 blockade. On the basis of this evidence, within the EO constituents, the principal chemical entity (*E*)-cinnamaldehyde was selected as chaperon for this action; hence, binding to CYP51 was assessed through dockings. The pose acquired highlighted some interesting features that might be in charge of the antifungal activity, mainly referring to the possibility of accommodating the whole (*E*)-cinnamaldehyde molecular scaffold in the middle of the lodge located over the heme group of the target, anchoring nearly perpendicularly by means of a coordination bond involving iron and the aldehydic carbonyl oxygen atom, as depicted in Figure 1.

This feature has been experimentally observed in the X-ray structure of other thiazoles in complex with different but functionally related cytochromes [49], as has the chelating ability of cinnamaldehyde also been proven by X-ray data diffraction [50,51]. In addition to this, π-π stackings of the Phe126 and Tyr118 aromatic rings and hydrophobic contact with the Ile121 and Ile304 sidechains handle the binding to the cavity dome of CYP51, as corroborated by favorable docking scores (see Section 4.2).

## 3. Discussion

The antifungal resistance of *Candida* spp. to azoles and echinocandins, the main therapeutic options for the treatment of candidiasis, continues to be an issue of global concern [58], along with biofilm formation [59]. Microbial biofilms are intricate, spatially organized communities of microorganisms ensconced within an extracellular matrix and adhered to a surface. *Candida* species are known to form intricate and dynamic biofilms consisting of diverse morphological manifestations of the fungus, including yeasts, pseudohyphae, and hyphae. According to the National Institutes of Health (NIH), more than 80% of human microbial infections are linked to biofilms [60]. *Candida* spp. biofilms play a significant role in recurrent and chronic infections, demonstrating resilience and/or resistance to diverse antifungal compounds and the innate immune system [61]. Due to the limitations in treating *Candida* spp. biofilms with conventional drugs, alternative options are urgently required. In this scenario, the drug repositioning approach, which aims to identify new therapeutic potential among already approved drugs, is part of an encouraging development in the field of antimicrobial drug discovery. The literature has reported that selective serotonin reuptake inhibitors (SSRIs) impact antimicrobial activity [11,38,62,63]. Sertraline (an SSRI), in addition to its primary purpose, exhibits antimicrobial activity against various yeast strains, either independently or when combined with antifungal agents [64,65]. Furthermore, effects against *Candida* spp. biofilms have been reported [39,66]. The precise mechanism by which sertraline acts is still not uniquely understood; however, evidence supports the hypothesis that sertraline, a serotonin reuptake pump inhibitor in humans, can also behave as an efflux pump inhibitor in bacteria [63]. In addition, some research corroborates the ability of sertraline to cause cell wall and membrane damage in *Candida* spp., also affecting ergosterol synthesis [14,67]. Another promising strategy for finding new treatments for acute fungal infections is the use of EOs in combination with traditional antifungal agents or repositioned drugs, known for their antifungal potential. This strategy aims to improve the treatment efficacy of conventional drugs and decrease toxicity, side effects, and resistance problems. Indeed, several EOs have been recognized as a significant reservoir of compounds suitable for application in fungal infections, especially in the treatment of biofilms [68,69,70]. The efficacy of these associations was substantiated by our previous studies related to the synergy of EOs with certain commercially available antimicrobials and diclofenac, a widely recognized non-steroidal anti-inflammatory drug, which displayed notable antimicrobial and antifungal efficacy, thereby supporting the potential therapeutic application of these combinations [35,36,71,72,73]. In addition, scientific studies reported synergistic activity of *C. verum* EO when used in combination with various antibiotics. Indeed, it was demonstrated that the synergistic combination of *C. verum* EO with piperacillin effectively tackled a beta-lactamase-resistant strain of *Escherichia coli*. One noteworthy finding revealed a remarkable two-fold reduction in the MIC of piperacillin, decreasing from 1024 µg/mL to 256 µg/mL, when used in association with the EO [74]. The capability of *C. verum* EO to cause irreversible membrane damage and to reduce the bacterial surface charge has been hypothesized as a plausible mechanism of action. One study also suggested the capability of *C. verum* EO to function as a quorum-sensing inhibitor, thus reversing *E. coli*’s resistance to piperacillin [75]. *C. verum* EO has been also demonstrated to enhance the antimicrobial activity of ampicillin or chloramphenicol against multi-resistant strains such as *Bacillus subtilis* and *Escherichia coli* [32]. More recently, we demonstrated the synergistic antibacterial effect of the non-antibiotic drug sertraline in combination with *C. verum* EO on several bacterial strains [36]. Besides its antibacterial potential, some studies have highlighted the synergistic activity of *C. verum* EO when combined with antifungal agents in addressing *Candida* infections. Indeed, it has been demonstrated that combined treatment of *C. verum* EO and fluconazole (a FIC value of 0.37) led to 69.51% killing of *C. albicans* cells after 3 h of incubation and allowed us to decrease the effective concentrations four- to eight-fold compared to the MIC values. Furthermore, this combination was found to inhibit ergosterol synthesis at sub-MIC values [76]. The same potent synergistic effect was demonstrated for the *C. verum* EO/amphotericin B association (FIC value of 0.37) against *Candida* spp. biofilm. Notably, this combination inhibited *C. albicans* biofilm formation with a percentage of 94%. This association has also been reported to affect the production of secreted aspartic proteases, a virulent factor for *C. albicans* ATCC 10231, while transcriptomic analyses demonstrated the combined effect of *C. verum* EO and amphotericin B in down-regulating Ras-cAMP-Efg and MAPK signaling genes and inhibiting the expression of adhesion and secreted aspartyl proteinase virulence factor genes [77]. Building upon these encouraging findings and what was achieved in our previous work, we aimed to extend our research to the evaluation of the antifungal effects of the association between *C. verum* EO and sertraline on different *Candida* spp. and their biofilms. As underlined in our in vitro assays, the fungal strains under study displayed sensitivity to the compounds tested, both individually and in combination, as shown in Table 1, thus confirming a clear-cut synergism between sertraline and *C. verum* EO. Particularly interesting was the significant reduction in the sertraline concentration needed for planktonic cells when it was used in combination with *C. verum* EO for all the yeast strains under study. A noteworthy decrease in sertraline’s active concentration was observed for *C. albicans* ATCC 90028, for which the MIC value of sertraline was found to decrease from 32.0 to 0.80 µg/mL. Meanwhile, for *C. albicans* ATCC 10231, *C. albicans* 10 A12, *C. glabrata* ATCC 15126, *C. kefyr* ATCC 204093, and *C. krusei* 31A29, the active concentration dropped from 64.00 to 3.20 µg/mL. These results are particularly interesting considering that these strains are responsible for most nosocomial infections of the bloodstream. The FICI values were significantly lower than the limit value of 0.5 for both the planktonic and sessile cells, thus confirming the substantial synergism between sertraline and *C. verum* EO. Indeed, the sertraline–*C. verum* EO combination considerably prevented the biofilm growth of all *Candida* spp. tested, with the inhibition percentages ranging from 59.90 to 86.44%. Also, in this case, it was possible to observe a significant decrease in the sertraline concentration necessary to inhibit biofilm growth when it was used in the combination. This outcome was particularly noteworthy in the case of *C. albicans* ATCC 90028. Indeed, while the use of sertraline alone necessitated 40.00 µg/mL, when combined with *C. verum* EO, a mere 4.00 µg/mL was adequate to achieve a substantial reduction in biofilm growth, by 86.4%. This clear synergistic effect was corroborated by a corresponding FICI value of 0.23, markedly lower than the threshold value of 0.5. The data also indicated a considerable synergistic effect on those strains from clinical isolation. Indeed, the concentration of sertraline combined with EO that was able to inhibit 69.44 ± 0.80% of *C. krusei* 31 A29’s biofilms amounted to 8.00 µg/mL, whereas sertraline alone necessitates 160.00 µg/mL. These data are particularly encouraging since the remarkable antimicrobial efficacy of this combination particularly stands out when compared to the associations between *C. verum* EO and the conventional antibiotics or antifungals reported in the literature, as documented by their lower FIC values. Moreover, the lower FIC values imply a more effective synergistic effect, whereby the combined action of this association achieves greater inhibition of microbial growth at lower concentrations than either compound alone. This is underscored by the considerable decrease in the concentration of the respective components within the association.

The effects achieved could be attributed to active substances in *C. verum* EO such as (*E*)-cinnamaldehyde, which is the major component, and others such as eugenol, caryophyllene, and cinnamyl acetate that are among the minor constituents, as reported in our previous work [37]. Our docking studies revealed that (*E*)-cinnamaldehyde binds effectively to the active site of CYP51, suggesting a potential mechanism by which this compound exerts its antifungal activity. This interaction is particularly significant as CYP51 is a key enzyme involved in the biosynthesis of ergosterol, an essential component of the fungal cell membrane. The inhibition of CYP51 can disrupt ergosterol production, leading to increased cell membrane permeability and ultimately cell death. This finding supports the hypothesis that (*E*)-cinnamaldehyde, as the principal constituent of *C. verum* essential oil, plays a major role in the observed antifungal effects. However, the mixture of these components present in *C. verum* EO conceivably contribute to the antifungal activity of the EO and work alongside with sertraline for the observed synergistic effect [78,79]. Indeed, it could be hypothesized that synergy arises from concerted action on multiple cellular targets. As previously reported, *C. verum* EO could act non-specifically against the cell envelope of microorganisms. Indeed, it has been reported that some of the constituents of *C. verum* EO, such as (*E*)-cinnamaldehyde and eugenol, could be responsible for damaging cell membrane integrity [80,81]. In addition, (*E*)-cinnamaldehyde could promote ROS generation in microbial cells, thus triggering cell death [82]. In this context, sertraline could act affecting by a specific target, such as ergosterol synthesis, or by hampering efflux pumps, which may, in turn, enhance the whole inhibitory effect. Otherwise, membrane permeabilization by *C. verum* EO’s components could enhance sertraline uptake [14,65,83].

## 4. Materials and Methods

### 4.1. Materials

#### 4.1.1. Essential Oil

The *C. verum* EO (Lot 140/0000324, 10.2018, 10 mL) was supplied by Erbe Nobili srl (Corato, Bari, Italy) and was stored in a vial shielded from light and at temperatures ranging from 0 to 4 °C until its use. The EO samples had already undergone gas chromatographic analysis (GC-MS) in our prior study [37].

#### 4.1.2. Chemicals

Sertraline was obtained from Sigma-Aldrich srl (Milan, Italy). The filters used were provided by Agilent Technologies Italia spa (Milan, Italy). The culture media employed were Sabouraud 2% dextrose broth (Oxoid, Rodano, Italy) and Yeast Malt Broth (Oxoid, Rodano, Italy).

#### 4.1.3. Fungal Strains

The antifungal activity was assessed against numerous fungal strains, encompassing various strains sourced from the American Type Culture Collection (ATCC, Rockville, MD, USA), which were *C. albicans* (ATCC 10231, ATCC 90028), *C. glabrata* (ATCC 15126), *C. tropicalis* (ATCC 750), *C. kefyr* (ATCC 204093), and *C. krusei* (ATCC 6258), and *Candida* strains derived from clinical isolation (*C. albicans* 10 A12, *C. albicans* A18, and *C. krusei* 31 A29). All the clinical isolates were obtained from patients admitted to the intensive care unit of the Department of Biomedical Sciences and Human Oncology, the University of Bari “Aldo Moro”, Italy. The isolation procedures were carried out in the hygiene section of the Department utilizing conventional physiological and morphological methods alongside an API system.

### 4.2. Methods

#### 4.2.1. Antifungal Testing

The strains were preserved at −80 °C in yeast peptone dextrose broth with 10–25% glycerol (Oxoid, Rodano, Italy) solution. Glycerol stocks were prepared for all the strains, stored at −20 °C, and subcultured on antimicrobial agent-free Sabouraud dextrose agar plates (BioMerieux, Marcy L’Etoile, France) to ensure their viability and purity before the start of the study. The antifungal activity of sertraline was assessed by means of the microdilution method, as described by the Clinical and Laboratory Standards Institute (CLSI, M27-A3) [84].

#### 4.2.2. Medium and Culture Conditions

Each frozen stock culture was inoculated in Sabouraud dextrose broth and incubated at 37 °C for 24 h in an orbital shaker at 60 rpm. Following incubation, the cells were transferred into a tube containing RPMI 1640 broth medium with L-glutamine and without bicarbonate buffered to a pH of 7 with MOPS, 3-(***N***-morpholino) propane sulfonic acid (165 M, Sigma, Milan, Italy). A standardized suspension of 1 × 10^6^ CFU/mL was obtained and immediately used.

#### 4.2.3. Preparation of the Test Solution

*C. verum* EO was dissolved in ethanol at a ratio of 1:5 and then further diluted in RPMI 1640 broth medium supplemented with Tween 80. Sertraline was appropriately dissolved in DMSO and subsequently in the culture medium (DMSO < 5%).

#### 4.2.4. The Checkerboard Test

The checkerboard method was employed to assess the synergistic, additive, or antagonistic effects of the combination of sertraline and *C. verum* EO. The tested dilutions were based on the MIC of the two substances. The combination of the two compounds was synergistic when the FICI was ≤0.5, additive when the FICI was >0.5 and <1, and antagonistic when the FICI was >1. The test was conducted using sterile 96-well microtiter plates containing sertraline and EO at two-fold serial concentrations. The MICs were obtained following incubation at 37 °C for 48 h. Each test was carried out in triplicate.

#### 4.2.5. Biofilm Biomass Measurement and Reduction

To assess the synergistic antibiofilm action of *C. verum* EO in association with sertraline, we conducted, in vitro, a colorimetric XTT (2,3-bis(2-methoxy-4-nitro-5-sulphophenyl)-5-[(phenylamino)carbonyl]-2*H*-tetrazolium hydroxide) assay. Briefly, 200 µL of yeast culture (10^6^ CFU/mL) was added to each well of a 96-well flat microtiter plate and incubated for 24 h at 37 °C by shaking it on a rocker table to facilitate cell attachment and biofilm formation. Subsequently, 200 µL of antibiotic was added as a positive control, while the negative control contained only RPMI 1640 without the sertraline–*C. verum* EO association. After incubation, the contents of each well were aspirated, and the wells were washed with 100 µL of sterile PBS to remove loosely adherent and nonviable cells. Following incubation, 200 µL of each sertraline–*C. verum* EO combination was added to the wells. Four double serial dilutions of the 40% ethanol *C. verum* EO with Tween 80 0.1% were prepared using the same method as described in our previous works for MIC evaluation. Dilutions of the *C. verum* EO were prepared alongside a series of double dilutions of the drug and the EO. This method ensured that all the sertraline dilutions were combined with appropriate concentrations of EO, resulting in a range of sertraline–EO concentration combinations [85]. These concentrations were prepared to represent 40%, 20%, 10%, and 5% of the MIC values for the EO and 25%, 12.5%, 6.25%, and 3.12% of the MIC values for sertraline [86,87]. Following incubation with the drug, the media was aspirated from each well, and the supports were washed with 1 mL of PBS. The biofilms were then plunged in 2 mL of PBS with the addition of 180 μL of XTT solution (1 mg/mL) and Menadione (0.4 mM) at a ratio of 6:1. This solution was prepared by dissolving XTT in sterile water (obtained by filtration) and Menadione in dimethyl sulfoxide. The plate with the supports was incubated for 2 h at 37 °C. After 2 h, the plate was read with a microplate spectrophotometer Tecan Infinite M1000 Pro multiplate reader (Tecan, Cernusco S.N., Italy) at a 490 nm wavelength, where there was a maximum Formazan absorbance peak. The sessile minimal inhibitory concentration (sMIC_50_) was determined as the lowest antibiofilm concentration for both compounds causing a visible reduction in the growth of the biofilm compared with the growth in the control well without the presence of sertraline or the EO. The percentage of the reduction in the biofilm obtained with the combinations studied was calculated using the following formula: percentage of biofilm reduction = (OD control well−OD experimental)/(OD control well) × 100 [31]. Antimicrobial susceptibility tests of the biofilms were performed in triplicate on different days. In our experimental protocols, the combinations of the substances were analyzed by calculating the FIC index (FICI). Generally, the FICI value was interpreted as (i) a synergistic effect when it was ≤0.5; (ii) an additive or indifferent effect when it was >0.5 or <1; and (iii) an antagonistic effect when it was >1 [88]. The results are expressed as means ± SDs of at least three distinct measurements performed in triplicate.

#### 4.2.6. Molecular Modeling Studies

The (***E***)-cinnamaldehyde SMILES string was converted into a three-dimensional structure and its energy relaxed with Open Babel [51] after 10,000 steepest descent iterations. The target protein (pdb code 5V5Z [52]) was prepared for docking with the Maestro Protein Preparation Wizard [53]. The binding poses were sampled throughout 1000 runs of the Lamarckian Genetic Algorithm (LGA) implemented in AutoDock 4.2.6 [54] using the GPU-OpenCL algorithm version [55], exploring a 85 × 85 × 85 Å^3^ box with its center on the iron atom and a spacing of 0.375 Å. The Gasteiger charges were calculated for the ligand and the heme group with QUACPAC [56] and the AMBER UNITED force field [57] for the peptide chain of CYP51. The best free energy of binding (−5.30 kcal/mol) and the most populated (1000/1000) pose were selected.

## 5. Conclusions

Recent research has shown promising outcomes in antimicrobial therapies that combine repositioned drugs with essential oils. Our previous study demonstrated the synergistic efficacy of *C. verum* EO and sertraline against various bacterial strains. Given the public health threat posed by *Candida* infections and the role of microbial biofilms in fostering resistance, our study aimed to assess the synergistic effects of sertraline and *C. verum* EO on *Candida* spp. in both planktonic and sessile states. Our results highlight the potent effect of this combination, significantly reducing the quantity of sertraline required to suppress fungal strains and decrease biofilm production. These results hold paramount importance since demonstrating the enhanced efficacy of the association at lower concentrations compared to the components taken individually, this novel combination offers a path towards more sustainable and effective treatment options. While our study was preliminary and conducted in vitro, further investigations are needed to clarify the mechanism underlying this synergism and to assess its clinical efficacy, particularly against biofilms on medical devices. These findings offer a promising foundation for exploring alternative antifungal therapies targeting biofilm infections and potentially overcoming the challenges associated with conventional antibiotics.

## Figures and Tables

**Table 1:** Chemical composition of *Cinnamomum verum* EO.

Compound	Peacks Area % ± SEM	Library/ID	SI/MS	LRI	AI
**1**	0.3 ± 0.1	α-Pinene ^a^	94	934	934
**2**	0.1 ± 0.03	Camphene	96	950	949
**3**	0.5 ± 0.1	β-Thujene	91	965	968
**4**	0.2 ± 0.1	α-Phellandrene ^a^	90	1005	1001
**5**	0.5 ± 0.1	p-Cymene	95	1021	1021
**6**	1.5 ± 0.30	Eucalyptol ^a^	98	1023	1023
**7**	7 ± 1	Linalool ^a^	97	1095	1098
**8**	0.2 ± 0.01	o-Anisaldehyde	98	1220	1222
**9**	73 ± 5	(*E*)-Cinnamaldehyde ^a^	97	1225	1226
**10**	0.2 ± 0.04	Safrole	97	1285	1287
**11**	0.4 ± 0.05	α-Cubebene	98	1348	1348
**12**	6 ± 1	Eugenol ^a^	98	1360	1359
**13**	4 ± 1	Caryophyllene ^a^	99	1410	1408
**14**	0.5 ± 0.1	Humulene	95	1452	1452
**15**	5 ± 1	Cinnamyl Acetate ^a^	97	1455	1455
**16**	0.2 ± 0.02	Eugenol Acetate	96	1525	1524
**17**	0.3 ± 0.04	Caryophyllene oxide	83	1580	1578
**18**	0.6 ± 0.1	Benzyl Benzoate	96	1755	1753

^a^: Standard compounds. The linear retention index (LRI) on an HP-5MS column was experimentally determined using a homologous series of C7-C30 alkane standard mixtures. The arithmetic index (AI) was taken from Adams 4th Ed. [40] and/or the NIST database [41]. Similarity indexes/mass spectra (SIs/MS) were compared with data reported in the NIST database and were determined as reported by Koo et al. [42] and Wan et al. [43]. Relative percentage values are means of three determinations with structural equation modelling (SEM) in all cases below 10%.

**Table 2:** Antifungal effect of sertraline (µg/mL), *Cinnamomum verum* EO (mg/mL), and their association on different *Candida* spp. strains.

Strains	MICo ^a^	MICc ^b^	FIC ^c^	FICI ^d^	R% ^e^
*C. albicans* ATCC 10231					
Sertraline	64.00	3.20	0.05	0.11	95
*C. verum* EO	0.32	0.02	0.06		94
*C. albicans* ATCC 90028					
Sertraline	32.00	0.80	0.03	0.08	97
*C. verum* EO	1.22	0.06	0.05		95
*C. albicans* 10 A12					
Sertraline	64.00	3.20	0.05	0.17	95
*C. verum* EO	0.64	0.08	0.12		87.5
*C. albicans* A18					
Sertraline	128.00	6.40	0.05	0.11	95
*C. verum* EO	0.32	0.02	0.06		94
*C. glabrata* ATCC 15126					
Sertraline	64.00	3.20	0.05	0.11	95
*C. verum* EO	0.64	0.04	0.60		94
*C. kefyr* ATCC 204093					
Sertraline	64.00	3.20	0.05	0.08	95
*C. verum* EO	0.32	0.01	0.03		97
*C. krusei* ATCC 6258					
Sertraline	64.00	6.40	0.10	0.16	90
*C. verum* EO	0.32	0.02	0.06		94
*C. krusei* 31A29					
Sertraline	64.00	3.20	0.05	0.11	95
*C. verum* EO	0.32	0.01	0.06		97
*C. tropicalis* ATCC 750					
Sertraline	128.00	12.80	0.10	0.16	90
*C. verum* EO	1.22	0.08	0.06		93

All the MIC (minimal inhibitory concentration) values for sertraline and *C. verum* EO are expressed in µg/mL and mg/mL, respectively. ^a^ MICo: MIC of single component tested alone; ^b^ MICc: MIC of each component in association at the most effective inhibition growth; ^c^ FIC: fractional inhibitory concentration; ^d^ FICI: fractional inhibitory concentration index; ^e^ R%: reduction percentage in the amount of each associated component compared to each single component. FIC and FICI are reported as the means of three replicates.

**Table 3:** Inhibitory effects (% Red) of *Cinnamomum verum* EO, sertraline, and their combinations on fungal biofilms.

		*C. verum* EO mg/mL		Sertraline µg/mL			Synergism	
Strains	sMIC_50_ ^a^	%Red ± SD ^b^	sMIC_50_ ^c^	%Red ± SD ^d^	*C. verum* EO mg/mL ^e^	Sertraline µg/mL ^f^	Sertraline + *C. verum* EO %Red ± SD ^g^	FICI ^h^
*C. albicans* ATCC 10231	0.80	52.90 ± 0.50	160.00	76.10 ± 0.82	0.05	16.00	72.8 ± 0.90	0.16
*C. albicans* ATCC 90028	3.05	67.25 ± 0.55	40.00	57.59 ± 0.50	0.40	4.00	86.44 ± 1.00	0.23
*C. albicans* 10 A12	1.60	55.16 ± 0.80	160.00	63.80 ± 0.80	0.20	16.00	75.29 ± 0.80	0.23
*C. albicans* A18	1.60	55.21 ± 0.70	320.00	53.07 ± 0.40	0.40	32.00	77.02 ± 0.40	0.35
*C. glabrata* ATCC 15126	0.8	83.09 ± 1.00	320.00	82.00 ± 1.00	0.10	16.00	59.90 ± 0.50	0.18
*C. kefyr* ATCC 204093	0.40	64.40 ± 0.45	320.00	84.80 ± 0.90	0.05	16.00	79.80 ± 0.90	0.20
*C. krusei* ATCC 6558	0.40	69.22 ± 0.20	320.00	67.43 ± 0.30	0.10	32.0	64.90 ± 1.00	0.35
*C. krusei* 31 A29	0.80	62.15 ± 0.50	160.00	65.27 ± 1.00	0.2	8.00	69.44 ± 0.80	0.30
*C. tropicalis* ATCC 750	1.52	55.30 ± 1.00	640.00	58.16 ± 0.80	0.38	32.00	68.25 ± 0.50	0.30

^a^ Sessile minimal inhibitory concentration of EO that reduced at least 50% of biofilm growth; ^b^ EO biofilm mass inhibition rate ± standard deviation; ^c^ sertraline sessile minimal inhibitory concentration that reduced at least 50% of the biofilm growth; ^d^ sertraline biofilm inhibition rate ± standard deviation; ^e^ concentration of the essential oil in the mixture at the most effective combination; ^f^ concentration of sertraline in the mixture at the most effective combination; ^g^ biofilm inhibition rate of the combination mixture; ^h^ fractional inhibitory concentration index; EO: essential oil.

## Data Availability

The data are contained within the manuscript.
